# e-Learning Evaluation Framework and Tools for Global Health and Public Health Education: Protocol for a Scoping Review

**DOI:** 10.2196/49955

**Published:** 2023-10-24

**Authors:** Awsan Bahattab, Michel Hanna, George Teo Voicescu, Ives Hubloue, Francesco Della Corte, Luca Ragazzoni

**Affiliations:** 1 Center for Research and Training in Disaster Medicine, Humanitarian Aid, and Global Health Novara Italy; 2 Department for Sustainable Development and Ecological Transition Università del Piemonte Orientale Vercelli Italy; 3 Department of Translational Medicine Università del Piemonte Orientale Novara Italy; 4 Research Group on Emergency and Disaster Medicine Vrije Universiteit Brussel Brussel Belgium

**Keywords:** computer-assisted instruction, disaster medicine, disaster, e-learning, educational assessment, global health, medical education, public health, evaluation, scoping, review methods, review methodology, education, educational

## Abstract

**Background:**

There has been a significant increase in the use of e-learning for global and public health education recently, especially following the COVID-19 pandemic. e-Learning holds the potential to offer equal opportunities, overcoming barriers like physical limitations and training costs. However, its effectiveness remains debated, with institutions unprepared for the sudden shift during the pandemic. To effectively evaluate the outcomes of e-learning, a standardized and rigorous approach is necessary. However, the existing literature on this subject often lacks standardized assessment tools and theoretical foundations, leading to ambiguity in the evaluation process. Consequently, it becomes imperative to identify a clear theoretical foundation and practical approach for evaluating global and public health e-learning outcomes.

**Objective:**

This protocol for a scoping review aims to map the state of e-learning evaluation in global and public health education to determine the existing theoretical evaluation frameworks, methods, tools, and domains and the gaps in research and practice.

**Methods:**

The scoping review will be conducted following the PRISMA-ScR (Preferred Reporting Items for Systematic Reviews and Meta-Analyses Extension for Scoping Reviews) guidelines. The initial search was performed in PubMed, Education Resource Information Center, Web of Science, and Scopus to identify peer-reviewed articles that report on the use of evaluation and assessment for e-learning training. The search strings combined the concepts of e-learning, public health, and health science education, along with evaluation and frameworks. After the initial search, a screening process will be carried out to determine the relevance of the identified studies to the research question. Data related to the characteristics of the included studies, the characteristics of the e-learning technology used in the studies, and the study outcomes will be extracted from the eligible articles. The extracted data will then undergo a structured, descriptive, quantitative, and qualitative content analysis to synthesize the information from the selected studies.

**Results:**

Initial database searches yielded a total of 980 results. Duplicates have been removed, and title and abstract screening of the 805 remaining extracted articles are underway. Quantitative and qualitative findings from the reviewed articles will be presented to answer the study objective.

**Conclusions:**

This scoping review will provide global and public health educators with a comprehensive overview of the current state of e-learning evaluation. By identifying existing e-learning frameworks and tools, the findings will offer valuable guidance for further advancements in global and public health e-learning evaluation. The study will also enable the creation of a comprehensive, evidence-based e-learning evaluation framework and tools, which will improve the quality and accountability of global health and public health education. Ultimately, this will contribute to better health outcomes.

**International Registered Report Identifier (IRRID):**

DERR1-10.2196/49955

## Introduction

Even before COVID-19, health systems around the globe were confronted with multiple challenges that necessitated strengthening health systems based on the primary health care approach and maintaining a good balance between public health and clinical care. The shortage in the health workforce, including public health professionals, is among the challenges that hinder the achievement of global health goals [1-3]. Still, the medical education literature addressing public health is scarce [4].

e-Learning (also known as digital education or technology-enhanced learning, among other synonymous terms) is a promising educational strategy to address the shortage of skilled health professionals through strengthening education [2]. The electronic-based approach to learning and education uses different forms of electronic devices, applications, or processes. The internet is the commonly used mode to deliver e-learning content, though non–internet-based delivery, such as via CD-ROM, audiotape and videotape, satellite broadcasts, stand-alone computers, and interactive televisions, are also being used [5,6].

In higher education, including medical education, the use of e-learning began in the 1990s and has been documented in the scientific literature since 2000 [7,8]. Structuring learning outcomes around clinical or public health competencies is a common approach to classifying and reporting e-learning interventions [2]. However, the usefulness and effectiveness of e-learning in medical education have long been debated [9-11]. Nevertheless, a well-designed and implemented e-learning program has the potential to provide equal opportunities for medical education by maintaining flexibility, and at the same time, overcoming multiple barriers to building health workforce capacity, such as physical barriers and training delivery costs [2,3,12].

More recently, the COVID-19 pandemic pushed education toward a distance structure and stimulated the exponential growth of e-learning education [8,13]. Unfortunately, many institutions around the globe were not prepared for the digital transformation [14,15]. Moreover, the effectiveness of e-learning depends on many factors that go beyond the e-learning intervention [16,17].

Hence, a pressing need to evaluate the different dimensions of e-learning interventions has emerged. To do so, a standard and rigorous approach to evaluating e-learning is essential. Existing evidence suggests that e-learning is associated with high satisfaction and improvement in knowledge and skills [18,19], and the effectiveness of e-learning for health professionals is comparable to that of traditional educational interventions. However, the existing literature in medical education is usually limited to measuring e-learning outcomes and comparing e-learning with traditional methodologies. Further research is needed, particularly focusing on its impact on trainees’ behaviors and patient outcomes [18,20].

Additionally, the validity of such evaluations is threatened by the dearth of literature that reports the use of standardized assessment tools [21,22] and theoretical foundations, which are essential to explain what and how learning can be facilitated [23-25]. Moreover, the methodological diversity among studies hampers the clear interpretation of the findings [26]. To establish a more definitive understanding of the benefits and limitations of e-learning in the health care sector, additional investigation and standardization of methods are essential.

The aim of the systematic scoping review will be to map the theoretical frameworks and models that explain the underlying concepts, domains, and constructs, as well as the assessment tools required, of rigorous e-learning evaluation. This will enable evidence-based practice as a means of ensuring accountability and validity in education.

It is worth noting that evaluation and assessment are often used interchangeably in the literature. In this paper, however, we will refer to evaluation as the process of obtaining information about any aspect of an educational program for subsequent judgment and decision-making. On the other hand, assessment will refer to the instruments that measure the learner's achievements, which comprise an essential component of the evaluation. To understand what is known about how public health e-learning is being evaluated, the scoping review will aim to identify and synthesize the available evidence on the evaluation frameworks and tools. The study will identify, describe, and discuss the theoretical frameworks and tools that are being used to evaluate public health e-learning. The PICO (Population, Intervention, Comparison, and Outcome) essential elements framework guided the development of the research questions (Table 1). Specifically, in the context of public health education, this study aims to answer the following questions: (1) How is e-learning evaluation conceptualized? (2) What are the existing theoretical models or frameworks to evaluate e-learning and what do they aim to evaluate? (3) How is e-learning evaluation investigated or measured? (4) Are there validated tools for evaluating e-learning? (5) What outcomes do these tools evaluate?

**Table 1:** PICO (Population, Intervention, Comparison, and Outcome) framework for the systematic scoping review.

Element	Description
Population	Health professionals or students
Interest	e-Learning evaluation and assessment frameworks and tools
Context	Public health and public health-related education
Outcome	Reporting the evaluation framework or tools

## Methods

The scoping literature review involved a systematic search following the PRISMA-ScR (Preferred Reporting Items for Systematic reviews and Meta-Analyses Extension for Scoping Reviews) checklist [27]. Unlike systematic reviews, which address a relatively narrow range of quality-assessed studies, a systematic scoping review addresses broad questions, mapping the key concepts underpinning a research area and the main sources and types of evidence with a range of methodologies, and does not undertake quality assessment.

### Inclusion and Exclusion Criteria

The articles selected in this review aim to describe or use a framework or tool to evaluate e-learning used for public health education. The e-learning target group could be undergraduate, graduate, or continuing education programs. All types of study designs of peer-reviewed, original studies that were published after 2000 in English will be included. See Textbox 1 for further details about inclusion and exclusion criteria.

**Inclusion and exclusion criteria.**

**Inclusion criteria**

- Population
  Includes health professionals or students at any level (undergraduate, graduate, or continuing education programs).
- Interest
  Articles addressing public health or public health–related fields (eg, global health, international health, One Health, planetary health, humanitarian health, disaster medicine or disaster management).
- Outcome
  Articles that describe evaluation frameworks or tools for e-learning.
- Article type
  Peer-reviewed original literature.
  Empirical and theoretical articles, including those with experimental and quasi-experimental study designs, descriptive and analytical observational study designs, and qualitative studies.
  Studies may or may not include comparison with conventional or other e-learning.
- Language, date, and availability
  Articles published after 2000.
  Articles written in English.
  Articles for which the full text is available.

**Exclusion criteria**

- Population
  The evaluated population did not include health professionals or students.
- Interest
  Articles that did not address public health or public health–related education.
  Articles focused on clinical skill evaluation.
  Articles focused on medical education topics related to individualistic clinical care, diagnostics, or basic health science.
- Outcome
  Articles that did not describe or use evaluation frameworks or tools.
- Article type
  Non–peer-reviewed grey literature.
  Secondary literature, such as reviews and meta-analyses.
  Opinion articles, theses, dissertations, book chapters, protocols, and editorials.
- Language, date, and availability
  Articles published before 2000.
  Non-English articles.
  Articles for which the full text is not available.

### Information Sources and Search Strategy

On January 31, 2022, a preliminary limited search of the PubMed database was conducted to identify relevant articles and keywords. On July 31, 2023, an updated search was conducted in the PubMed, Web of Science, Education Resource Information Center, and Scopus databases for studies published from January 1, 2000, onward. The search was limited to the English language. Search terms included the concepts of e-learning, public health, and health science education and evaluation, frameworks, and tools using “text word searching,” which involves searching for a word or phrase appearing anywhere in the document using Boolean operators and truncations (Table S1 in Multimedia Appendix 1). 

### Study Selection and Screening

The identified records will be imported into Rayyan [28] to streamline the screening process. Duplicates will be removed before titles and abstracts are reviewed independently by 2 authors for the inclusion criteria. We will use the standard PRISMA flow diagram to provide the study selection process. The articles included in the review must report the use of evaluation or assessment for e-learning training within the specified inclusion criteria for the population, topics, and level of education. After initial review, the reviewers will go through the complete text and apply the inclusion criteria.

### Data Extraction and Data Items

Data from eligible studies will be extracted by 2 reviewers. The following data will be extracted and charted from each paper: (1) descriptive data of the included study profile, (2) study characteristics data, (3) e-learning and technology characteristics, and (4) the study outcome, including evaluation framework, evaluation methods, and evaluation tools (Table S2 in Multimedia Appendix 1). The data extraction tool will be tested and may be adjusted and amended during the process, and any changes will be documented in the final report. The summary table will improve transparency and reproducibility by showing what types of data were extracted from which studies.

### Analysis

The data will be synthesized using structured, descriptive, quantitative, and qualitative content analysis of the main themes and an overview will be provided for the current scope of the literature. In addition, the research team will analyze findings regarding the studies’ overall purpose and evaluate the implications for future research, practice, and policy.

### Ethical Considerations

No ethical board approval is necessary to conduct this scoping review.

## Results

**Figure 1:**
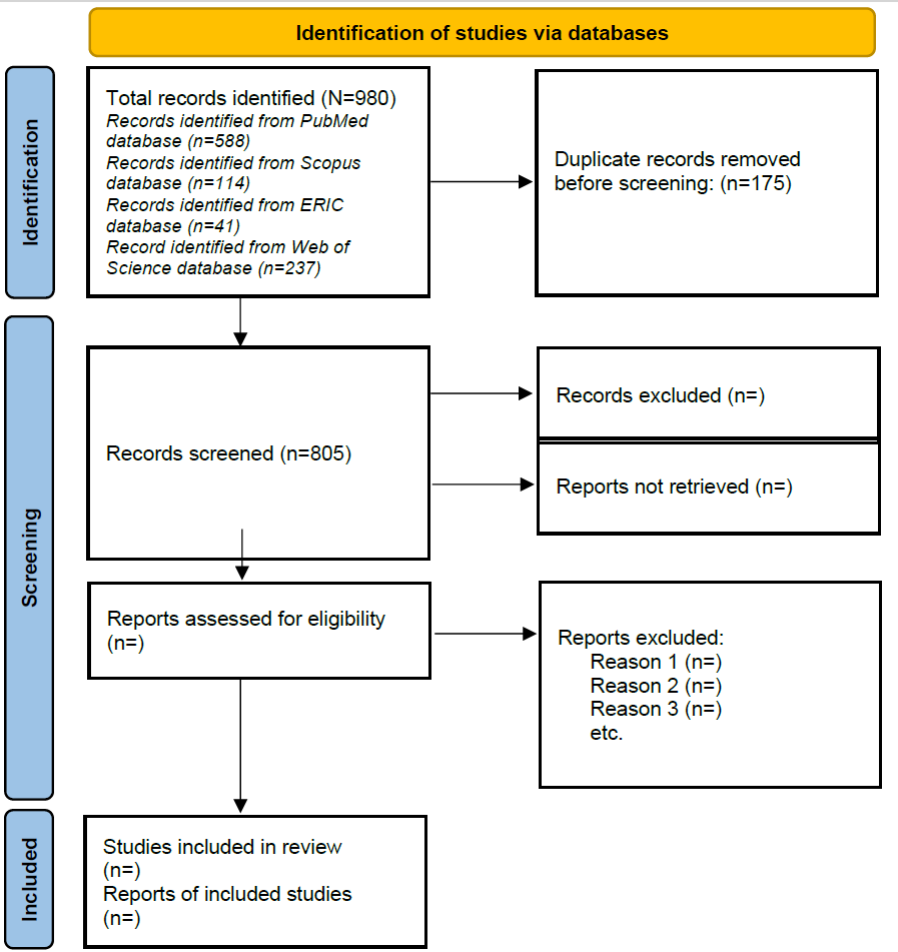
Flowchart diagram of the study selection process based on PRISMA (Preferred Reporting Items for Systematic Reviews and Meta-Analyses).

Targeted searches were conducted in January 2022 to inform the development of a comprehensive search strategy for electronic database searches. This strategy was iteratively developed for and tested in PubMed. Iterative refinements to the scoping review protocol and formalization of the methods were completed by July 2022 and updated on July 31, 2023. The final search was conducted in the PubMed, Scopus, Education Resource Information Center, and Web of Science databases. The initial database searches revealed 980 studies. The database searches were completed in August 2022, duplicates were removed, and title and abstract screening of 805 extracted articles is currently underway. Study selection, data extraction and analysis, and drafting of the manuscript to report the results of the scoping review will be conducted throughout 2023. Any changes to the methods reported here will be documented and reported. The PRISMA flowchart will be used to describe the research selection procedure (Figure 1), and the scoping review’s findings relevant to the study objectives will be presented.

## Discussion

### Expected Findings

Comprehensive multifaceted evaluation of e-learning in the context of public health and global health training is essential to make an evidence-based decision about the future investment in health workforce capacity building. This scoping review is expected to identify existing evaluation frameworks and tools for evaluating public health e-learning.

To the best of our knowledge, this will be the first systematic scoping review to address public and global health as a growing field of medical education. While previously published reviews did not focus on medical education [29] or were limited in scope [29-31], this study will identify the theoretical framework, methods, and domains commonly used in the evaluation of public and global health e-learning. Thus, the study will underline the existing gaps in the scope and practice of e-learning evaluation.

A previous systematic review for e-learning evaluation identified 8 themes [29]. Most of these studies focused on only one or two aspects. Moreover, the representation of evaluation themes varied among different disciplines, technologies, and educational levels. Some of these themes were applied more than others, while others were underrepresented [29]. Still, previous reviews lacked or underrepresented information about public health and global health education [1]. Moreover, the proliferation of e-learning and related literature in the past few years necessitates reviewing the current status of the literature in the field. In addition, previous systematic reviews revealed that educational theories and theoretical learning frameworks rarely guided e-learning evaluation, raising concerns about the quality and validity of training evaluation.

### Strengths and Limitations

The review will adhere to a robust methodology following the recommended standards for conducting scoping reviews [27,32] that allows transparency and replicability.

The limitations of scoping reviews, which also apply to this review, will be noted. The literature search was applied to 4 relevant search engines. Due to limited time and resources, the search was restricted to English and peer-reviewed articles. Hence, some articles may be missed from the review. Since the scoping review aims to understand the status of evaluating e-learning rather than measuring effectiveness, we will not use a quality appraisal or bias assessment for the included studies.

### Future Directions

The lack of comprehensive and robust guidance on e-learning evaluation is an obstacle for educators to ensure the quality and accountability of public health e-learning [3,33]. Hence, the results of this review will allow public health and medical educators to understand how public health e-learning is being evaluated from theoretical and practical points of view. The discussion will reflect on the current status of e-learning evaluation in public health education and compare the findings to the existing body of knowledge. Moreover, the results can guide the development of an evidence-based, field-specific, multifaceted, integrated model for e-learning evaluation and recommend the most appropriate evaluation methods, and tools, as well as domains to be evaluated.

### Conclusion

In conclusion, this review will enhance our knowledge about the current practice of e-learning evaluation in global and public health education. The findings will inform the development of a comprehensive field-specific evaluation framework and tools, with the ultimate aim of improving the quality and accountability of global and public health e-learning.

## Appendix

Multimedia Appendix 1 Supporting information regarding the search strategy and data items for extracting information from eligible articles.

## Abbreviations

PICO: Population, Intervention, Comparison, and Outcome
PRISMA-ScR: Preferred Reporting Items for Systematic Reviews and Meta-Analyses Extension for Scoping Reviews

## Data Availability

All data generated or analyzed during this study are included in or alongside this article.
