# Supplementary material for: e-Learning Evaluation Framework and Tools for Global Health and Public Health Education: Protocol for a Scoping Review
Source: JMIR Res Protoc. 2023 Oct 24;12:e49955. doi: 10.2196/49955 (PMC10630868; doi:10.2196/49955)
Supplement: Multimedia Appendix 1 [file resprot_v12i1e49955_app1.doc]

**Supplementary file:**

**Table S1: Search strategy**

| **Search terms and search queries for PubMed**  PubMed via the National Library of Medicine  **Search run 31/01/2022** | | | | |
| --- | --- | --- | --- | --- |
| **Search** | **Search term** | **Query** | **Item found** | |
| **#1** | **E-Learning** | "eLearning" OR e-Learning OR "electronic learning" OR "online learning" OR "computer-based learning" OR "computer-based instruction" OR "computer-assisted instruction" OR "virtual environment" OR "virtual scenario" OR "virtual simulation" OR "virtual learning" OR "virtual learning environment" OR "virtual reality" OR "online education" OR "technology-enhanced learning" OR "distance education" OR "online course*" OR "distance learning" OR "m-learning" OR "mobile learning")) | 42,783 results | |
| **#2** | **Theoretical Framework** | ((education) AND (evaluation OR assessment) AND ("model" OR "framework" OR "tool" OR "instrument" OR "questionnaire" OR "test" OR "survey")) | 311,532 results | |
|  | Educational measurement tool |  |  |  |
| **#3** | **Health Sciences Education** | **"**health science*" OR "medical education**"** “one health” OR “global health” OR” public health” OR “international health” OR “epidemiology” OR “disaster medicine” OR “disaster management” OR “humanitarian aid” OR “humanitarian assistance” ( NOT (clinical)) NOT (radiology)) NOT (surgery)) NOT (ambulatory)) | 845,618 results | |
| **#4** | **e-learning evaluation** | #1 AND #2 | 7,845 results | |
| **#5** | **e-learning evaluation in health sciences** | #3 AND #4 | 459 results | |
| **#6** |  | #5 and Filters: Full text, English, from 2000/1/1 - 2022/01/31 Sort by: Publication Date  Search: (("eLearning" OR e-Learning OR "electronic learning" OR “digital learning” OR "online learning" OR "computer-based learning" OR "computer-based instruction" OR "computer-assisted instruction" OR "virtual environment" OR "virtual scenario" OR "virtual simulation" OR "virtual learning" OR "virtual learning environment" OR "virtual reality" OR "online education" OR "technology-enhanced learning" OR "distance education" OR "online course*" OR "distance learning" OR "m-learning" OR "mobile learning") AND (((education) AND (evaluation OR assessment) AND ("model" OR "framework" OR "tool" OR "instrument" OR "questionnaire" OR "test" OR "survey")))) AND ((("health science*" OR "medical education" "one health" OR "global health" OR "public health" OR "international health" OR "disaster medicine" OR "disaster management" OR "humanitarian aid" OR "humanitarian assistance") ) NOT (clinical OR radiology OR surgery OR ambulatory)) Filters: Full text, English, from 2000/1/1 - 3000/12/12 Sort by: Publication Date | 447 results | |
| **Search repeated on 31/07/2023** | | | | |
|  |  | Search: (("eLearning" OR e-Learning OR "electronic learning" OR "online learning" OR "computer-based learning" OR "computer-based instruction" OR "computer-assisted instruction" OR "virtual environment" OR "virtual scenario" OR "virtual simulation" OR "virtual learning" OR "virtual learning environment" OR "virtual reality" OR "online education" OR "technology-enhanced learning" OR "distance education" OR "online course*" OR "distance learning" OR "m-learning" OR "mobile learning") AND (((education) AND (evaluation OR assessment) AND ("model" OR "framework" OR "tool" OR "instrument" OR "questionnaire" OR "test" OR "survey")))) AND ((("health science*" OR "medical education" "one health" OR "global health" OR "public health" OR "international health" OR "disaster medicine" OR "disaster management" OR "humanitarian aid" OR "humanitarian assistance") ) NOT (clinical OR radiology OR surgery OR ambulatory)) - Saved search Filters: Full text, English, from 2000/1/1 - 3000/12/12 Sort by: Most Recent | 588 | |
| **Search queries for database other than PubMed (31/07/2023)** | | | | **Item founds** |
| **Scopus** | | | | |
| ( TITLE-ABS-KEY ( "eLearning"  OR  "digital learning"  OR  "e-Learning"  OR  "electronic learning"  OR  "online learning"  OR  "computer-based learning"  OR  "computer-based instruction"  OR  "computer-assisted instruction"  OR  "virtual environment"  OR  "virtual scenario"  OR  "virtual simulation"  OR  "virtual learning"  OR  "virtual learning environment"  OR  "virtual reality"  OR  "online education"  OR  "technology-enhanced learning"  OR  "distance education"  OR  "online course*"  OR  "distance learning"  OR  "m-learning"  OR  "mobile learning" )  AND  TITLE-ABS-KEY ( ( education )  AND  ( evaluation  OR  assessment ) )  AND  TITLE-ABS-KEY ( ( "model"  OR  "framework"  OR  "tool"  OR  "instrument"  OR  "questionnaire"  OR  "test"  OR  "survey" ) )  AND  TITLE-ABS-KEY ( ( "health science*"  OR  "medical education"  "one health"  OR  "global health"  OR  "public health"  OR  "international health"  OR  "disaster medicine"  OR  "disaster management"  OR  "humanitarian aid"  OR  "humanitarian assistance" ) ) )  AND  ( EXCLUDE ( SRCTYPE ,  "p" )  OR  EXCLUDE ( SRCTYPE ,  "k" ) )  AND  ( EXCLUDE ( PUBYEAR ,  1998 )  OR  EXCLUDE ( PUBYEAR ,  1996 ) )  AND  ( LIMIT TO ( LANGUAGE ,  "English" )  OR  EXCLUDE ( LANGUAGE ,  "German" )  OR  EXCLUDE ( LANGUAGE ,  "Italian" ) ) | | | | 114  Results |
| **Web of science** | | | | |
| (eLearning) OR (computer-based learning) OR (virtual learning) (Topic) and (one health) OR (global health) OR (public health) OR (international health) OR (disaster medicine) OR (disaster management) OR (humanitarian aid) OR (humanitarian assistance) (Topic) and (evaluation OR assessment) (Topic) not clinical OR radiology OR surgery OR ambulatory (All Fields) and Safety Training (Exclude – Search within topic) and Article (Document Types) and 2022 or 2021 or 2020 or 2019 or 2018 or 2017 or 2016 or 2015 or 2014 or 2013 or 2012 or 2011 or 2010 or 2009 or 2008 or 2007 or 2004 or 2003 or 2002 or 2001 (Publication Years) and English (Languages) | | | | 237 results |
| **ERIC** | | | | |
| ( "Digital learning" or "eLearning" OR e-Learning OR "electronic learning" OR "online learning" OR "computer-based learning" OR "computer-based instruction" OR "computer-assisted instruction" OR "virtual environment" OR "virtual scenario" OR "virtual simulation" OR "virtual learning" OR "virtual learning environment" OR "virtual reality" OR "online education" OR "technology-enhanced learning" OR "distance education" OR "online course*" OR "distance learning" OR "m-learning" OR "mobile learning")) ) AND ( higher education or university or college ) AND ( evaluation or assessment ) AND ( framework or model or theory or approach or tool ) AND ( (evaluation OR assessment) AND ("model" OR "framework" OR "tool" OR "instrument" OR "questionnaire" OR "test" OR "survey")) ) AND ( "health science*" OR "medical education" “one health” OR “global health” OR” public health” OR “international health” OR “epidemiology” OR “disaster medicine” OR “disaster management” OR “humanitarian aid” OR “humanitarian assistance”  **Limiters** - Peer Reviewed; Date Published: 20020101-20211231; Journal or Document: Journal Article (EJ); Education Level: Higher Education  **Expanders**- Apply equivalent subjects  **Search modes** - Boolean/Phrase | | | | 41 results |

**Table S2: Initial list of data items for extracting information from eligible articles**

| **Data items** |
| --- |
| 1. **Study Profile** |
| Authors |
| Title |
| Country of the study |
| Year of publication |
| 1. **Study Characteristics** |
| Study Design |
| Study subject/topic |
| Study Population |
| 1. **E-Learning Characteristics** |
| Mode of Access |
| Modality |
| Communication |
| Instruction methods and strategies |
| 1. **Study Outcome** |
| ***Theoretical evaluation framework /model*** ***description*** |
| ***Evaluation tool(s)*** |
| Numbers of reported evaluation/ assessment tool (s) |
| The evaluation tool(s) |
| Evaluation/ assessment tool (s) validation |
| Evaluation/ assessment tool (s) outcome(s) |
